# You can count on the motor cortex: Finger counting habits modulate motor cortex activation evoked by numbers

**DOI:** 10.1016/j.neuroimage.2011.11.037

**Published:** 2012-02-15

**Authors:** Nadja Tschentscher, Olaf Hauk, Martin H. Fischer, Friedemann Pulvermüller

**Affiliations:** a Medical Research Council, Cognition and Brain Sciences Unit, 15 Chaucer Road, Cambridge, CB2 7EF, UK; b Department of Psychology, University of Potsdam, 14476 Potsdam OT Golm, Germany; c School of Psychology, The University of Dundee, Dundee, DD1 4HN, UK

**Keywords:** Embodied cognition, Numerical cognition, Finger counting habits, SNARC effect

## Abstract

The embodied cognition framework suggests that neural systems for perception and action are engaged during higher cognitive processes. In an event-related fMRI study, we tested this claim for the abstract domain of numerical symbol processing: is the human cortical motor system part of the representation of numbers, and is organization of numerical knowledge influenced by individual finger counting habits? Developmental studies suggest a link between numerals and finger counting habits due to the acquisition of numerical skills through finger counting in childhood. In the present study, digits 1 to 9 and the corresponding number words were presented visually to adults with different finger counting habits, i.e. left- and right-starters who reported that they usually start counting small numbers with their left and right hand, respectively. Despite the absence of overt hand movements, the hemisphere contralateral to the hand used for counting small numbers was activated when small numbers were presented. The correspondence between finger counting habits and hemispheric motor activation is consistent with an intrinsic functional link between finger counting and number processing**.**

## Highlights

►Individual finger counting habits modulate motor cortex activation to numbers. ►Small digits activate motor areas contralateral to the hand used in finger counting. ►Number processing is grounded in individual experience related to finger counting. ►Abstract symbol processing is embodied in perceptual-motor brain networks.

## Introduction

Theories of “embodied cognition” assume that sensory-motor processes are a fundamental element of human cognition. This view has received much recent support from both behavioral and neuroscientific investigations (e.g. Barsalou, 2008). Numerical concepts are of particular interest when investigating the validity of embodied cognition because numbers are traditionally considered as prototypical instances of abstract symbol representations without remaining links to earlier sensory or motor activation (Piaget, 1942). In this traditional view, the concept of “four” would be derived by generalizing over numerous instances of encountering sets of four objects and following an understanding of the principles of one-to-one correspondence, stable ordering, order irrelevance, and cardinality (Gelman and Gallistel, 1978). Proponents of embodied cognition have pointed out that systematic sensory-motor activities during number acquisition remain part of our numerical knowledge (e.g. Lakoff and Núñez, 2000). Specifically, most humans in most cultures learn to count on their fingers while they count objects and acquire simple number concepts (Butterworth, 1999; Lindemann et al., in press). A persistent linkage of number symbols with specific actions due to manual-verbal counting activity could be explained by Hebbian learning mechanisms.

Similarly, in the language domain, Pulvermüller (2001) proposed that cortical connections between motor and language circuits are strengthened whenever concepts relating to actions are acquired. More specifically, “Hebbian learning” based on correlated neuronal activity serves as a general explanation for the formation of distributed functional circuits in the brain and accounts for embodied cognition signatures of symbols which are manifested in both neural activation and overt behavior (Hebb, 1949). This view has been supported by neuroscientific studies on action-words (Hauk et al., 2004; for recent review, see Pulvermüller and Fadiga, 2010), as well as at the behavioral level (for recent review, see Fischer and Zwaan, 2008; Glenberg and Kaschak, 2002). Most importantly for the present study, the motoric meaning of action related words is reflected at the sentence processing stage even in idiomatic expressions such as “grasping an idea”, thus indicating that embodied semantics contributes to the comprehension of abstract sentence meaning (Boulenger et al., 2009). In the field of numerical cognitions, Sato and Lalain's (2008) results support Hebbian learning mechanisms due to manual-verbal counting activities. They found that a majority of people overtly verbalize numbers during finger counting. They investigated finger counting strategies in four different age groups of 4–47 years old participants and showed that such tendency to verbalize numbers during finger counting did not differ between groups.

The idea that number concepts are embodied leads to specific behavioral and neuroscientific predictions. Behaviorally, there should be an effect of finger counting experience on numerical cognition. This prediction was supported by several studies. Developmental work supports the view that knowledge about numbers is built up by counting with the fingers (cf. Fayol et al., 1998; Gracia-Bafalluy and Noel, 2008; Noel, 2005). For adults, Fischer (2008) took the association between small numbers and left space (the “spatial-numerical association of response codes” or SNARC effect (Dehaene et al., 1993)) as a starting point to compare people who start counting with their left hand (left-starters) against those who start counting with their right hand (right-starters). Left-starters showed a robust SNARC effect, with faster left-hand responses to small numbers in a parity classification task, but there was no consistent spatial bias for the group of right-starters, presumably reflecting the conflict between their finger counting habits and the typical left-to-right increasing number lines (cf. Shaki et al., 2009). This is in line with Di Luca et al. (2006) who tested finger-digit response compatibilities in a reaction-time paradigm and demonstrated that a mapping congruent with prototypical finger-counting strategies leads to better performance than a mapping congruent with a left-to-right oriented mental number line (cf. Di Luca and Pesenti, 2008, 2010). Thus, finger counting habits modify the association between numbers and space in adults. The fact that finger gnosis predicts arithmetic skills in 5–6 years old children provides further evidence that the use of fingers during the acquisition of numerical knowledge has a causal influence on the learning process itself (Noel, 2005). By using either canonical finger gestures or vertical bars to present results of simple arithmetic problems in a naming task, Badets et al. (2010) demonstrated that even in adults simple arithmetic operations are still unconsciously based on finger-numerical representations. Further support that gestures and finger movements reveal implicit numerical knowledge and enhance learning of maths comes from studies investigating the influence of forced gestures on arithmetic task performance. Encouraging children to gesture during arithmetic problem solving enhances their performance significantly as compared to groups in which either no attention was directed to gesturing at all, or children were told to keep their hands still (Broaders et al., 2007; Cook et al., 2008). Moreover, children who were told to gesture whilst solving arithmetic tasks benefited more from subsequent math lessons on new problems compared to a group that was instructed not to move their hands in a previous training on math problems. The authors concluded that telling children to gesture encourages them to convey previously unexpressed implicit ideas which makes them receptive to instructions of new problems and, in turn, enhances learning. Together, these results suggest an important role of body actions in arithmetic processing.

Still, it remains to be shown that specific numerical concepts are grounded in specific actions of the body. Do grounded concepts (for example individual finger counting habits) shape the way in which numerical knowledge is organized? The aim of this study was to investigate whether numerical concepts related to finger counting habits are represented in the human cortical motor system. Neuroscientifically, it has been established that single-number perception involves predominatly the parietal lobes bilaterally, and in particular the horizontal intraparietal sulci (in line with the triple-code model by Dehaene and Cohen, 1995; 1997; Dehaene et al., 2003). Additionally, numerical processes such as simple and complex mental calculation involve regions known to be activated in a broad variety of cognitively challenging tasks (multiple-demand network, Duncan (2010)). In a meta-analysis, Arsalidou and Taylor (2011) show that across fMRI studies, the prefrontal cortex including the inferior frontal sulcus, the anterior cingulate cortex, the precentral gyrus and the insula were significantly active in all four basic arithmetic tasks. Further, the embodied view of numerical cognition predicts that the processing of number concepts activates motor systems of the brain (Butterworth, 1999; Gallese and Lakoff, 2005), as supported by a recent body of neuroscientific studies (Andres et al., 2007; Pesenti et al., 2000; Sato et al., 2007; Zago et al., 2001; for recent review, see Michaux et al., 2010). The regions of interest in this context include sensorimotor cortex in the pre- and postcentral gyrus and central sulcus (Brodmann areas BA 1–4 and 6) along with inferior frontal areas in and adjacent to Broca's area (BA 44 and 45). Evidence for a link between numbers and sensory-motor based finger representations comes from neuropsychological patients with Gerstmann syndrome who exhibit both dyscalculia and finger agnosia (together with dysgraphia and left-right confusion; Ardila et al. (2000); for recent review, see Rusconi et al. (2010)). Testing the prediction of motor cortex activation during numerical processing in healthy participants, Sato et al. (2007) measured changes of excitability of hand muscles during performance of visual parity judgment tasks on numerals 1 to 9 when TMS was applied to hand motor cortex. They found an increase in amplitude of motor-evoked potentials for right hand muscles during the presentation of small numbers (1 to 4) in right-handed participants. Recruitment of participants for the current study revealed that, among right-handed people, individuals who start counting with the right hand, thus assigning small numbers to the right hand's fingers, are approximately ten times more common than left-starters (see below). Sato et al.'s (2007) results indicate that shared neuronal networks for numerals and finger movements are mediated by people's individual finger counting habits (cf. Andres et al., 2007). This supports the idea that finger-counting strategies influence number processing in adults, against the argument that numbers only build up on a finger-based representation through bottom-up processes (Andres et al., 2008). A strict proof of this claim does, however, require a systematic evaluation of left- and right-starters' behavior and brain activation.

In summary, recent studies have shown that finger counting habits contribute to numerical knowledge and arithmetic ability in children and adults. However, evidence that motor cortical activations specifically reflect counting habits, as embodied manifestations of abstract cognitive activity, is still lacking. The aim of this study was to investigate if hand-related motor and premotor cortex (BA 4 and 6) become active when symbols related to number concepts are processed, and whether such motor system activation is systematically linked to finger counting habits. Specifically, for left-starters activation for numbers 1–5 was expected to appear in the right motor cortex and vice versa for right-starters.

## Methods

### Participants

Data from 29 participants (15 females; 14 males) entered the final analysis. They were all right-handed monolingual native speakers of English, and reported no left-handed family members. They had normal or corrected-to-normal vision and no history of neurological or psychiatric disorder. Data from three participants had previously been excluded because of unacceptable head movements within the scanning sessions or lack of hand preference. In addition, participants were pre-screened according to their finger counting habits by using a finger counting questionnaire (Supplementary Material, Fig. 1). The questionnaire was tested in an independent study on a group of 21 healthy, right-handed participants. Observation protocols of participants' finger-counting habits were taken and compared with results of the questionnaire, confirming its precision in selecting left- and right-starters. As reported by Fischer (2008, p. 389) this questionnaire has good reliability and face validity, and this has recently been confirmed in Lindemann et al. (in press). Two groups of 15 right-starters (7 females; 8 males) and 14 left-starters (8 females; 6 males) entered the final analysis, for which lateralization of neural activation was compared in a between-subject design. Further, all selected participants in this study started counting with their thumb and continued with the index, middle and ring finger until they reached their little finger. The mean age of participants was 25 years (right-starters: 25.1; left-starters: 24.7; SD for all participants: 5.2; SD right-starters: 5.4; SD left-starters: 4.8). Hand preference was confirmed by a ten-item version of the Edinburgh handedness inventory (mean Laterality Quotient for right-starters: 80; SD: 32.6; for left-starters: 82; SD: 20.5) (Oldfield, 1971). Participants received £20 for their participation. Ethical approval was obtained from the Cambridge Local Research Ethics Committee.

### Materials of imaging experiment

Numerical stimuli consisted of 10 Arabic digits (0 to 9) and the corresponding number words (“zero” to “nine”) which were presented in separate blocks. Two blocks contained Arabic digits, the other two blocks the number words. Numbers and number words were repeated 21 times within each block, resulting in 210 trials. The maximum height of number stimuli was 15 mm and their width was less than 4 degrees of visual angle when written in Calibri font (12 points). In order to control for effects of stimulus complexity, neither the number “10”, as a two-digit number, nor the corresponding number word was included. In addition to digits and number words, 40 baseline stimuli were presented within each block: two different single Greek letters (“μ” and “ς”) were intermixed with Arabic digits; corresponding letter strings (“ςςςς” and “μμμμ”) were intermixed with the number words. The length of the single Greek letters matched the length of the presented Arabic digits, measured in millimeters when presented on the screen. The length of the Greek letter strings was varied in order to match the length of the different number words. Finally, each block contained 5% “attention-trials”, used to keep participants' attention during the experiment. For the purpose of this task, single letters of the English alphabet were presented intermixed with the Arabic digits. Pronounceable pseudo-words like “ons” or “soat” were chosen for blocks containing number words. Participants were requested to perform a foot pedal response as soon as they detected one of those trials with the foot of their choice, but were asked to use the same foot all the time. A foot-response was chosen because motor cortical activations for foot and finger movements were assumed to be clearly distinct. Hence, foot-responses during the attention task should not affect the hypothesized motor cortical activation for finger movements during numerical processing. Overall, 210 Arabic digits versus number words, 40 baseline stimuli, and 10 attention-trials were presented within each block. Considering the four blocks of stimulus presentations, this resulted in a total of 1040 trials.

Two different finger localizer tasks were performed after the four blocks of Arabic digits and number words in order to measure peaks of activation for individual finger movements within hand motor cortex areas. In the first task (finger-localizer), participants saw instructions on the screen that referred to each individual finger (for example “left thumb”, “right index finger”, “left middle finger”). They were requested to alternately move the corresponding finger up and down for as long as its name stayed on the screen, at a rate of approximately one movement per second and to rest their fingers when the word “rest” was presented on the screen. In the second task (counting-localizer), the Arabic digits 1 to 9 were presented on the screen. Participants were asked to count repeatedly from 1 up to the presented number in their usual finger counting habits and to rest their fingers when a “0” was presented on the screen.

### Procedure of imaging experiment

Stimuli were presented in a randomized order by means of E-prime software 2.0 (Schneider et al., 2002) and viewed via a back-projection screen located in front of the scanner and a mirror placed on the head coil. The main experiment was run in 4 blocks of approximately 10 minutes duration. Afterwards the two localizer tasks were performed for ten minutes each. Digits and number words were visually presented in an event-related design for 100 ms each with a fixation-cross in-between, which was presented with a jittered SOA of 1800–2100 ms.

Participants were requested to keep their attention during the whole experiment and to press the foot-pedal as soon as they detected one of the attention-trials. This instruction was repeated before each of the four blocks of stimulus presentations. Additionally, participants were told not to move their head and body and especially not their hands during the presentation of digits and number words (exception was made for their foot when they had to respond to attention-trials). They were naïve with regard to our experimental questions but received further information after the experiment.

In the finger-localizer task, each cue type was presented 5 times for ten seconds each in randomized order. During the counting-localizer task the numbers 0 to 10 were presented 5 times for ten seconds each in sequential order. To demonstrate the counting-localizer task, a short movie was presented before the task started that showed the movements for numbers 1 to 3. Two different versions of this movie were used in order to present the task to right-starters and left-starters in their respective finger counting habits.

### Materials and procedure of SNARC-test

The SNARC effect (cf. Fischer, 2008) was measured for each participant outside the scanner to assess the link between spatial-numerical associations and finger counting habits. The results were used as covariates for part of the fMRI analysis. The experiment was run on a 37 × 27 cm CRT monitor using E-Prime software (Schneider et al., 2002). Participants sat on a height-adjustable chair in front of the screen. The stimuli consisted of Arabic digits (1 to 9) (size: 15 × 10 mm; font type: Calibri) that were randomly presented, colored in white on a black background in the center of the screen. Responses were recorded on the “A” and “6” keys of an extended QWERTY keyboard with numerical keypad and delivered with left and right index fingers, respectively. The experiment had two blocks, each consisting of 10 practice trials followed by 90 experimental trials. Numbers were presented in random order ten times each per block, preceded by a fixation cross that was shown on the screen for 1 s. Each digit remained on the screen until a response was recorded. Participants were instructed to decide whether a presented number was odd or even (parity judgment task) by pressing one of two buttons as soon as the digit appeared on the computer screen. The response rule (even digit — left hand or even digit — right hand) was switched after the first block and the order of response rules was counterbalanced across participants. A beep tone was played for incorrect responses.

### Imaging methods

Participants were scanned in a 3-T Siemens (Munich, Germany) Tim Trio magnetic resonance system using a head coil. Echo-planar imaging (EPI) sequence parameters were TR (inter-scan interval) = 2 s, TE = 30 ms and flip angle = 78°. The functional images consisted of 32 slices covering the whole brain (slice thickness 3 mm, inter-slice distance 0.75 mm, in-plane resolution 3 × 3 mm). Imaging data were processed using SPM5 software (Wellcome Department of Imaging Neuroscience, London, UK; http://www.fil.ion.ucl.ac.uk/spm).

Images were realigned, coregistered, normalized and finally smoothed. This sequence of pre-processing steps was automated using software tools developed at the Cognition and Brain Sciences Unit (http://imaging.mrc-cbu.cam.ac.uk/imaging/AutomaticAnalysisManual). During the realignment process images were corrected for spatial movements and slice-timing, interpolating images in time to the middle slice using sinc interpolation. The EPI images were coregistered without skull stripping to the structural T1 images by using a mutual information coregistration procedure focused on intra-subject differences: images for the same subject from different scanning sessions were matched in space. The structural MPRAGE MRI (256 × 240 × 160, 1 mm isotropic) was normalized to the 152-subject T1 template of the Montreal Neurological Institute (MNI). The resulting transformation parameters were applied to the coregistered EPI images. During the spatial normalization process, images were resampled with a spatial resolution of 2 × 2 × 2 mm^3^. Finally, all normalized images were spatially smoothed with a 10-mm full-width half-maximum Gaussian kernel. A similar sequence of processing steps was applied to the motor localizer data.

Single-participant statistical contrasts were computed by using the general linear model based on the canonical hemodynamic response function (Friston et al., 1998). Low-frequency noise was removed with a high-pass filter (time constant 128 s for stimuli-conditions; 200 s for motor localizer). Each stimulus type (Arabic digits and number words “1” to “9”, baseline stimuli, and attention-trials) was modeled as a separate event type, i.e. as separate columns of the design matrix. Timing onsets for each event type were extracted from E-Prime output-files using Matlab. For digit and number word conditions, the first two scans in each session were excluded as well as trials preceded by an attention-trial, comprising a “dummy-scan” variable which was included in the design matrix. Group data were analyzed with random-effects analyses.

Contrasts for events were defined on a single-subject level first and later included in the random-effects analysis for group statistics. Because the digit “0” as well as the corresponding number word was not relevant for the main hypotheses, these stimuli were excluded from the following contrasts. All digits and number words together were contrasted against the combined baselines of their conditions (i.e., all Greek symbols), yielding the contrast “combined number words and digits > baseline” (referred to as “numbers > baseline” from now on). Similarly, all digits and number words were separately contrasted against their respective baselines, which resulted in the contrasts “digits > baseline” and “number words > baseline”. Digits and number words “1” to “5” and “6” to “9” were contrasted against baseline, yielding the contrasts “digits (1–5) > baseline”, “number words (1–5) > baseline”, “combined number words and digits (1–5) > baseline” (referred to as “numbers (1–5) > baseline” from now on), as well as the same contrasts for the numerals 6 to 9, respectively. Direct comparisons such as described above were also carried out between pairs of symbol types.

For the localizer tasks each single magnitude presented in the finger- and the counting localizer was contrasted against its respective “rest”-condition. Additionally, all finger counting gestures and single finger movements of each hand were separately contrasted against baseline.

### Data analysis: main approach

Whole-brain data were analyzed using SPM5. Regions of interest (ROIs) were defined and analyzed using the Marsbar utility in SPM5 (Brett et al., 2002). Activation referring to representations of Arabic digits and number words was investigated in primary and pre-motor cortical areas according to three questions: Is motor cortex activated by numbers? Is motor cortex activation lateralised according to groups for small numbers in line with participant's counting habits? Does this lateralization depend on numerical magnitude (i.e. large versus small numbers)? The following steps of data analysis were performed: First, activation from motor localizer tasks was analyzed. In order to define regions of interest, activation peaks were localized for the entire group (left plus right starters taken together). Due to the block design of the motor localizer tasks, strong activation patterns could be observed in sensory-motor regions. Therefore the FWE significance threshold of 0.05 was chosen for this analysis. Peaks of activation were used in a Small Volume Correction (SVC) for contrasts of digits and number words. This was done in order to investigate whether processing of numerical stimuli activated motor cortical areas. Second, lateralization of activation in motor cortical areas in line with participants' finger-counting habits was investigated. An hypothesis-guided SVC was conducted for precentral gyrus. Using an anatomical mask, FDR-corrected significant peaks of activation were extracted for an ROI analysis testing differences in hemispheric lateralization between left- and right-starters. Additionally, activation in those regions was explored further by using the lower threshold of p(unc) < .001. Note that in the above analyses, the contrasts used to define ROIs were independent of those computed for further analysis.

#### Investigation of primary motor cortical activation

Activation in motor cortex was explored according to the question as to whether activation for numerical stimuli appears in the same region as peaks of activation observed during finger movements. For this purpose, a whole brain analysis was conducted for each stimulus-condition and the localizer tasks, for the whole group of subjects. In addition, activation of the stimulus conditions was investigated for the groups of left-starters and right-starters separately. A spherical Small Volume Correction (SVC) with a radium of 15 mm was conducted for the contrast “numbers > baseline” using coordinates of the finger- and counting-localizer tasks. As in previous studies on action-word activation in motor cortex, we did not expect our peaks of activation to numbers to coincide exactly with the peaks in our localizer task (e.g. Hauk et al., 2004). Instead, we used SVC to allow for some variability around the localizer coordinates, while still controlling for multiple comparisons.

#### Effects of lateralization in motor cortex

Our main hypotheses are with respect to finger-counting related lateralization of activation in motor cortical areas, i.e. we expected an interaction Lateralization-by-Counting Habits. We therefore localized peaks of activation for “numbers (1–5) > baseline” in left and right motor areas, based on SVC around peaks in the motor localizer task (see above). This resulted in reliable peaks in the contralateral hemispheres of the corresponding starter groups, but only weak activation in the ipsilateral hemisphere. We therefore ran two different ROI analyses, in order to address the problem of a possible bias of our results due to ROI selection. First, we used the ROI in the left-hemisphere based on right-starter results, and in the right hemisphere based on left-starter results, as a factor Laterality in our ANOVA analysis. Assuming that the localization of motor areas is similar for left- and right-starters (note that both groups were right-handed), but that statistical sensitivity for the ipsilateral activation is low, this would be an unbiased selection of ROIs to detect an interaction Starter Group * Laterality (in other words: the right starters are used to detect left-hemispheric motor ROIs for both right- and left-starters, and vice versa for right hemisphere ROIs). In a second analysis, we weakened this assumption by choosing separate ROIs for left- and right-starters in both hemispheres. ROIs for right-starters were chosen at the peak activation for numbers in the left hemisphere, and at the symmetrical location in the right hemisphere. The reverse logic was applied to left-starters. Parameter estimates were subjected to ANOVAs evaluating the within-subject factors “Laterality” (left versus right hemisphere) and the between-subject factor “Counting Habits” (left versus right-starters). In addition, two-tailed t-tests against zero were conducted in order to analyze activation within each hemisphere and counting group separately.

#### SNARC effect as a possible confound

Direction and strength of the SNARC effect were assessed by calculating the average difference between correct reaction time (RT) of the right hand minus correct RT of the left hand for each participant and each digit shown. This score was then regressed on the digit magnitude, yielding for each participant a non-standardized regression weight which captured the direction and strength of the spatial mapping of numbers (Fias et al., 1996; cf. Fischer, 2008). These regression coefficients were tested against zero within each group and against each other between groups. Individual SNARC scores were included as covariates in ROI analyses.

## Results

### Behavioral results and SNARC effect

For the attention task, foot pedal responses were analyzed for each participant individually. Across participants, this resulted in 97% hits, 7% false-positives and 3% misses. No participant had more than three missed trials per session (corresponding to 30% of all required responses).

For the SNARC post-test, we found an overall significant regression weight for both groups of − 7.05 ms/digit (SD 9.00), t(27) = 4.14, p < .001. The effect was significant for left-starters with − 9.04 ms/digit (SD 8.32), t(12) = 3.91, p = .002 and for right-starters with − 5.32 ms/digit (SD 9.49), t(14) = 2.17, p = .047. The statistical comparison between groups yielded no reliable difference, t(26) = 1.09, p = .284, and the variances in the two groups also did not differ significantly, F(26) = 0.08, p > .05. In conclusion, despite a trend consistent with Fischer's (2008) finding, the present data did not show significant differences in strength and direction of the SNARC effect between the two groups. Hence, it was unlikely that spatial-numerical association effects confounded the imaging data. We still included the SNARC scores as co-variates in some of our analyses below.

### Whole brain analysis

**Fig. 1:**
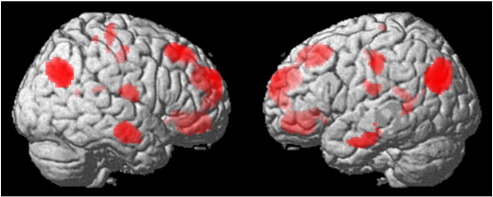
Illustration of activation for number words and digits, which were together contrasted against baseline and rendered on a standard brain surface. Threshold: p(FDR) < .05.

Whole brain data were explored using an FDR-corrected significance threshold of 0.05 (Fig. 1). The contrast “number words > baseline” showed more widespread activations than the contrast “digits > baseline” (Table 1). Peak activations for number words separately, as well as collapsed over digits and number words, occurred mainly bilaterally in the parietal cortex (BA 39) as well as in frontal areas (BA 9, BA 10 and BA 11), where stronger activation was found in the right hemisphere. The right superior temporal cortex (BA 22) showed activation for the contrast of combined digits and number words. For this contrast, the left inferior temporal cortex (BA 20), the left medial temporal cortex (BA 48), and the left anterior cingulate cortex (BA 32) showed activation as well. For the contrast “number words > baseline”, activation was found in the right posterior cingulate cortex (BA 23). Numbers contrasted against baseline showed activation in left medial-temporal areas (BA 48) as well as in bilateral parietal cortex (BA 39), right frontal cortex (BA 9 and 10) and left primary somatosensory cortex (BA 3).

### Investigation of motor cortical activation

An SVC was conducted on coordinates from the finger-localizer task. Activation peaks were localized at a family-wise error corrected (FWE) significance threshold of 0.05. For the contrast “numbers > baseline” significant right-hemispheric activation for the group of left-starters was found (p(FWE) = .035). No activation above threshold was found for coordinates in the left hemisphere, suggesting lateralization effects in line with finger counting habits in primary motor cortical areas for the group of left-starters. However, no activation above threshold could be observed in the group of right-starters at all. A smaller overlap, when compared to finger localizer-results, was found for peak coordinates of activation taken from the counting-localizer task in the group of left-starters. The SVC revealed no significant results. Again, no overlap of activation was found in the group of right-starters.

### Effects of lateralization in motor cortex

**Fig. 2:**
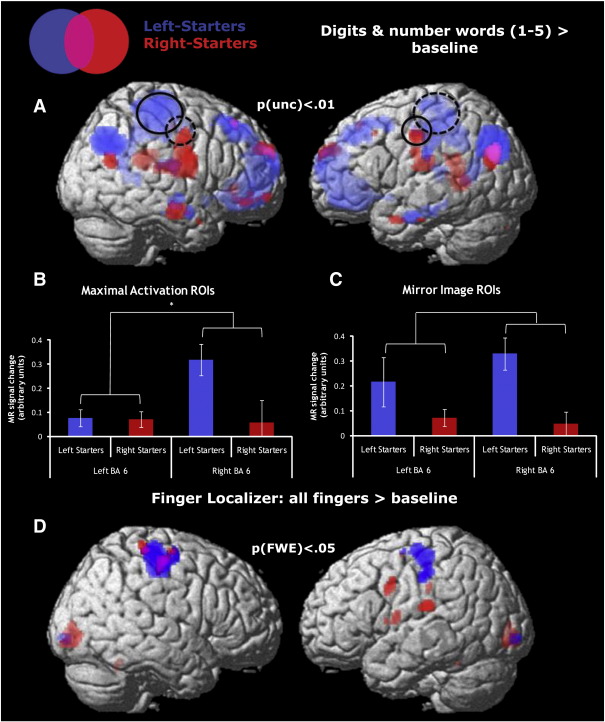
Activation of Left and Right-Starters in precentral gyrus (BA 6). (A) Illustration of haemodynamic activation in premotor cortex rendered on a standard brain surface. Red color referring to activation of right-starters, blue color presenting activation of left-starters. Contrast “numbers (1–5) > baseline”; threshold: p(unc) < .01. Solid-lined circles indicate main regions of activation for the respective counting groups [30, − 26, 62; − 22, − 14, 50], dotted-lined circles the mirror images of these regions [22, − 14, 50; − 30, − 26, 62]. (B) The graph illustrates mean parameter estimates (in arbitrary units) for cluster differentially activated by subgroups of right-starters (red) and left-starters (blue) in premotor cortex. Contrast “numbers (1–5) > baseline”; the significant counting group-by-region interaction is shown by brackets. (C) The graph illustrates mean parameter estimates (in arbitrary units) for cluster differentially activated by subgroups of right-starters (red) and left-starters (blue) in premotor cortex, with mirror images of extracted regions. Contrast “numbers (1–5) > baseline”; the marginal significant counting group-by-region interaction is shown by brackets. (D) Illustration of haemodynamic activation of finger-localizer, rendered on a standard brain surface. Red color referring to activation of right-starters, blue color presenting activation of left-starters. Contrast “all fingers > baseline”; threshold: p(FWE) < .05.

In this hypothesis-guided analysis, finger-counting-pattern related effects of hemispheric lateralization were investigated using ROIs in precentral gyrus (see Fig. 2, panel A). A Small Volume Correction (SVC), using a bilateral anatomical mask of precentral gyrus (Automated Anatomical Labeling (AAL) of activations (Tzourio-Mazoyer et al., 2002)), yielded significant activation for left-starters in right premotor cortex p(FDR) = .021. Based on peaks of activation for left-starters in this region, one ROI [30, − 26, 62] was extracted using data from the whole brain analysis for the contrast “numbers (1–5) > baseline”. No significant activation was found for the SVC in the left hemisphere. For right-starters, a peak of activation was found in left premotor cortex [− 22, − 14, 50] for the same contrast of “numbers (1–5) > baseline”, p(unc) = .001 (not significant at FDR threshold). The contralateral mirror image of these regions was defined as well to rule out that lateralization effects were restricted to regions of peak activation from the whole brain data (see section *Confirmation with mirrored ROIs* below).

Finger-counting habit dependent effects of hemispheric lateralization were investigated by subjecting parameter estimates to ANOVAs evaluating the within-subject factor “Laterality” (left versus right hemisphere) and the between-subject factor “Counting Habits” (left versus right-starters). In line with our hypothesis, significant effects of hemispheric lateralization were found for both counting groups: the ANOVA evaluating the factors “Laterality” and “Counting Habits” revealed a significant interaction Laterality-by-Counting Habits for the three contrasts “digits (1–5) > baseline”, “number words (1–5) > baseline” and “numbers (1–5) > baseline” (F(1,27) = 4.51, p = .043, F(1,27) = 4.62, p = .041, and F(1,27) = 7.64, p = .010, respectively) (see Fig. 2, panel B). As a control condition, the same ANOVAs were run with the numbers “6” to “9”. No significant interactions were found for these numerals. When including the SNARC-scores of individual participants as covariate, the same small digit interactions tended to be even stronger; the corresponding statistics were: F(1,26) = 5.93, p = .022, F(1,26) = 5.33, p = .029, and F(1,26) = 9.98, p = .004, respectively. These results indicate hemispheric lateralization effects for the two counting groups in line with their respective finger counting habits. In addition, two-tailed t-tests against zero were run for numerals “1” to “5” and “6” to “9” within each counting group and hemisphere. They confirmed activations contralateral to the starting hand in each counting group for the contrast “digits (1–5) > baseline” (left-starters (t(13) = 3.56, p = .003 in right hemisphere; right-starters t(14) = 3.13, p = .007 in left hemisphere). No such effects were found in the hemispheres ipsilateral to the “starting hand” in either group. No activation was found for the contrast “numbers (6–9) > baseline” in premotor cortical areas contralateral to the starting hand. Together, the results suggest significant effects of lateralization in both left and right starters only for small numerals (“1” to “5”).

#### Confirmation with mirrored ROIs

In this analysis, as described above, peak coordinates of activation in contralateral precentral areas for each group were applied to their respective group only, and mirror images of the extracted coordinates were used in order to define regions in the ipsilateral hemisphere. For left-starters this yielded a left-lateral region in posterior-dorsal premotor cortex [− 30, − 26, 62], for right-starters a right-lateral region in dorsolateral premotor cortex [22, − 14, 50]. The two-way ANOVA with the factors “Laterality” and “Counting Habits” revealed a marginally significant interaction Laterality-by-Counting Habits for the contrasts “numbers (1–5) > baseline” and “number words (1–5) > baseline”; F(1,27) = 3.44, p = .074 and F(1,27) = 3.73, p = .064, respectively (Fig. 2, panel C). Inclusion of SNARC-scores yielded similar results for the contrasts “numbers (1–5) > baseline” and “number words (1–5) > baseline”; F(1,26) = 3.81, p = .062 and F(1,26) = 4.04, p = .055, respectively. No effects were found for ANOVAs with the contrast “number (6–9) > baseline”. This confirms our hypothesis that the pattern of hemispheric lateralization is only present for small numerals. In line with these effects, a two-tailed *t*-test against zero revealed significant left-hemispheric activation for small numerals in the group of right-starters (t(14) = 3.13, p = .007), and significant right-hemispheric activation in the group of left-starters (t(13) = 3.568, p = .003), whereas no significant activation was found for the same contrast in the contra-lateral hemisphere of each group. An independent sample *t*-test confirmed significant differences between groups in the right hemisphere (t(27) = 3.546, p = .001), and marginally significant group-differences in the left hemisphere (t(27) = 1.728, p < .090). Overall, analyses with mirrored ROIs confirmed the pattern of lateralized activation for small numerals in line with individual finger counting habits. It is important to note that counting-group related general differences in activation for combined number words and digits were in line with differences in activation pattern for finger movements (Fig. 2, panel D).

Finally, the co-variation of individual results in the SNARC test with activation in the motor system was investigated. No significant correlation between activation in analyzed regions within the motor cortex (BA 4, BA 6) and individual SNARC scores were found in neither of the counting groups.

## Discussion

This study investigated the embodied basis of number processing, analyzing fMRI activation in cortical motor areas evoked by Arabic digits and number words. Most importantly, we found that laterality of cortical activation reflected the participants' finger counting habits, i.e. whether they preferred to start counting with their right or left hands. The group of right-starters revealed left-hemispheric activation for small numerals, and the group of left-starters showed right-hemispheric activation. This means that, when perceiving small numerals or small number words, the hemisphere controlling the hand which would be used during counting shows relatively enhanced activation, despite the absence of overt counting behavior.

### Interpretations of lateralized motor cortical activation for finger-counting groups

Activation for small numerals, lateralized according to individual finger counting habits, was found in the precentral gyrus. Small numerals mainly activated left-lateral premotor cortical regions in the group of right-starters, and right-lateral premotor cortical areas in the group of left-starters. For the first time, this study presents data of haemodynamic activation indicating that the cortical representation of numbers is modified by individual finger counting habits. These findings are open to several interpretations which we consider in turn.

First, recent research on numerical processing suggests a shared neural network for number processing and planning of finger movements, including parietal cortical areas, as well as precentral gyrus and primary motor cortex (Butterworth, 1999). In such a network, number perception might elicit the sub-threshold tendency to move associated fingers. The magnitude processing of numerals, as well as the planning and control of finger movements, is driven by parietal areas, which subsequently activate precentral gyrus and primary motor areas, in order to perform the planned finger movements (for review see Butterworth, 1999; Rusconi et al., 2005). The anatomical overlap of neuronal activation in parietal and precentral areas for numerical processing and performance in simple arithmetic tasks, as well as grasping movements and pointing, has been confirmed by several neuroimaging studies (Andres et al., 2007; Pesenti et al., 2000; Simon et al., 2002, 2004; Zago et al., 2001), which inspired the “neuronal recycling hypothesis” of cortical maps (Dehaene and Cohen, 2007). According to this hypothesis a shared neural network results from invasion of evolutionarily older brain circuits by more recent cultural inventions like the number system, which, in turn, inherit many of their structural constraints.

Second, the association between numbers, as well as number words and individual finger counting movements, during individual development of numerical skills in childhood would be predicted based on a “Hebbian learning” approach to semantic circuits (Pulvermüller, 1999): Because children use their fingers during counting and while solving simple counting problems, neuronal activation for processing of numbers and the movement of fingers becomes correlated. Although most adults do not count on their fingers as often as during childhood, such neuronal circuits due to early co-occurrence of neuronal activity for numbers/number words and finger movements might still exist in later life. In support of this, the most common and well-replicated finding of bringing numbers into a spatial organization (SNARC-effect) seems to be modulated by individual finger-counting habits in adults (Fischer, 2008).

We now discuss, in light of the present data, the resulting predictions from the “neuronal recycling” and the “Hebbian-learning” hypotheses, for the specific role and interaction of motor and parietal cortices in numerical processing.

### General differences between counting groups

We found less activation for right-starters compared to left-starters across all contrasts in motor cortical areas in the current study. When conducting a Small Volume Correction with coordinates from the finger-localizer task, significant activation in primary motor regions (located contralateral to their starting-hand) was only found in the group of left-starters. However, in premotor cortex both, right-starters and left-starters showed activation for numerals 1–5 contralateral to their starting-hands, but not in ipsilateral regions, suggesting lateralization of activation in line with their counting habits. This means that small numerals activate more strongly in the hemisphere contra-lateral to the respective “starting-hand” of each group. One explanation for generally weaker motor cortical activation in right-starters might be that over-learned motor patterns yielded less stimulus-specific activation. Because all subjects were right-handed, it is likely that they use their right hand for more tasks and skills than the left hand. This might lead to reduction of activation for a specific task in left hand motor cortex, responsible for execution of right hand movements, in all tested participants. Hence, for the group of right-starters, who was expected to show left-lateralized motor cortical activation in response to small numbers, this could explain weaker counting-related hemispheric lateralization effects (as compared to left-starters), because most actions with the dominant hand are highly familiar and over-learned. Surprisingly, premotor cortical regions showed effects of hemispheric lateralization in line with finger-counting habits for both counting groups. This supports the assumption that planning of finger counting movements (in premotor cortical areas) rather than their execution might have an important impact on numerical representations in adults.

This raises the question of how the link between numbers and finger counting habits builds up during childhood. The laterality of counting habits revealed by the present study, is in line with the prediction of the Hebbian learning hypothesis about the association of symbols and motor movements in semantic processing (Pulvermüller, 2001). It appears that the frequent co-occurrence of lateralised finger counting when processing small numerals and digits provides a suitable account for the lateralised brain activation of premotor/motor cortex when only the symbols are being presented, but any overt response is discouraged. Classically, – in the sense of a perceptual mirror mechanism (cf. Di Pellegrino et al., 1992; Rizzolatti, 1998) – such a connection between semantics and sensory-motor concepts was explained by children's observation of others, who introduced the relevant concept and performed the corresponding movement at the same time (Pulvermüller and Fadiga, 2010). However, in the case of numbers, self-execution of finger counting habits might be the most relevant supporting mechanism when building up a number concept, rather than observation mechanisms as previously discussed in the context of the mirror neuron system. If such general mirror mechanisms had been crucial, the close link between counting habit and symbol processing laterality and the interactions observed would be more difficult to explain.

Furthermore, eye movements have been shown to be modulated by number processing and arithmetic task solving (Fischer et al., 2004; Knops et al., 2009), and could therefore be considered as a possible confound of our results. However, previous studies reported eye-movement related activation in precentral areas (e.g. the frontal eye field) that are clearly distinct from those found in the current study. In a meta-analysis on regions involved in eye movements, attentional shifts and gaze perception, Grosbras et al. (2005) reported activation in precentral gyrus (e.g. [44, 4, 46] in Talairach space for attentional shifts in the right hemisphere; corresponding to [44, 2, 50] in MNI space) that were more anterior to our coordinates (e.g. [30, − 26, 62] in MNI space), which were found close to activation peaks of the hand-movement localizer. It would also be difficult to explain the lateralized activation patterns in line with participants' finger counting habits on the basis of eye movements. We conclude that eye movements cannot account for our results, and are not a serious confound for our conclusions.

It is important to note that such group differences were not mediated by the SNARC effect. There was no difference between counting groups in this fMRI study with respect to the SNARC effect, which can therefore not be considered as a confound of our results. In support of this, no significant correlations between activation in motor cortex and individual SNARC-scores were found in either group. Inclusion of SNARC-scores as a covariate in ANOVAs did not change the qualitative pattern of our results. This indicates that no confounding effect of the measured SNARC-scores on the current set of data existed. However, the statistical power for detecting finger-counting-habit related group differences may have been too low in this study compared to Fischer (2008), who found significant differences between SNARC scores of 53 left-starters and 47 right-starters.

### Motor cortex and cognitive functions

Various studies have revealed that the premotor cortex is involved in non-motor functions, which might be crucial for numerical processing. In an fMRI-study, Kansaku et al. (2007) showed that the upper part of the left ventral premotor cortex is specifically activated during counting of large stimulus sequences. In the same study the crucial role of this area in counting of large sequences (more than 20 trials) was confirmed by TMS, which disrupted participants' counting ability when it was applied to the left ventral premotor cortex. Moreover, activation was found in premotor areas during the performance of addition-tasks with single-digit numbers. In Hanakawa et al.'s (2002) fMRI-study, people silently added numerals between 1 and 9 and then verbally reported the result. Hanakawa et al. (2003) compared mental operation tasks including single-digit addition with silent verbal rehearsal tasks including seven-digit numbers. They found the anterior and dorsal part of the lateral premotor cortex significantly more activated for mental operations than for verbal rehearsal, whereas the ventral part was similarly active in both tasks. Compared to this, activation in the current study was found in posterior-dorsal parts of the right premotor cortex as well as in the left dorsolateral premotor cortex. Zago et al. (2001) compared simple multiplication tasks (single-digit numbers) with complex multiplication (two-digit numbers) in a PET-study, investigating the premotor cortex' role in arithmetic fact retrieval versus active mental calculation. The left ventral premotor cortex was activated in both tasks. Jost et al. (2009) compared multiplication tasks with small single-digit operands with those containing larger single-digit operands in an fMRI-study. They found increased activation in the anterior cingulate cortex and premotor cortex specifically for tasks with larger operands. According to these authors, such premotor cortical activation reflects increased conflicts during fact-retrieval processes, as well as higher demands for controlling and coordinating multiple processing steps in complex arithmetic problems. Such task-complexity dependent activation of precentral gyrus in arithmetic processing was confirmed by several other recent fMRI studies (cf. Fehr et al., 2007; Gruber et al., 2001; Menon et al., 2000). These studies provide evidence that the premotor cortex is involved in various higher cognitive processes including numerical manipulation tasks and arithmetic. Hence, it may mediate the transition from motor to cognitive functions. The causal relationship between motor cortex and number processing should be further elucidated by future TMS studies (for recent review, see Sandrini and Rusconi, 2009). Studies using methods with high temporal resolution, i.e. EEG or MEG, could reveal the time course of these processes, which is important to distinguish early automatic from later strategy-dependent processes (e.g. Hauk et al., 2008).

In conclusion, our study revealed that the human cortical motor system is involved when we perceive numerals. Moreover, lateralization of activation in premotor cortex evoked by numbers is modulated by individual finger counting habits. In line with behavioral, developmental and neuropsychological studies, our results provide evidence for the role of premotor cortex in number processing, as suggested by theories of embodied cognition.

The following is the supplementary material related to this article.

Fig. 1 Finger counting questionnaire.

## Figures and Tables

**Table 1:** Whole-Brain results for number words and digits contrasted against baseline. MNI coordinates and SPM5 group statistics of most strongly activated voxels for the contrasts “number words > baseline”, “digits > baseline” and “numbers > baseline”. Regions, that are false-discovery rate (FDR) 0.05 corrected significant, are marked with an asterisk.

Contrast	Label	Cluster			Voxel				MNI		
Brodmann's area	p(cor)	k	p(unc)	p(FWE)	p(FDR)	p(unc)	T	x	y	z
Words - baseline	L parietal (39)*	0.007	452	0.001	0.010	0.008	0.000	6.32	− 50	− 74	30
R prefrontal (11)*	0.000	1716	0.000	0.035	0.008	0.000	5.78	8	34	− 12
	0.000	1716	0.000	0.139	0.009	0.000	5.15	2	48	− 8
L prefrontal (11)*	0.000	1716	0.000	0.072	0.008	0.000	5.45	− 8	34	− 12
R post cingu (23)*	0.065	246	0.011	0.339	0.013	0.000	4.70	10	− 54	22
L inf temp (20)*	0.913	22	0.408	0.689	0.025	0.000	4.22	− 56	− 14	− 26
R mid temp (21)*	0.584	69	0.146	0.770	0.029	0.000	4.11	64	− 10	− 20
L front eye field (8)*	0.626	63	0.164	0.855	0.034	0.000	3.97	− 24	24	50
Numbers - baseline	L mid temp (48)	0.128	485	0.007	0.679	0.360	0.000	4.33	− 42	− 18	20
L parietal (39)	0.391	307	0.027	0.817	0.360	0.000	4.13	− 46	− 80	28
R parietal (39)	0.551	248	0.043	0.878	0.360	0.000	4.02	44	− 76	38
R dor lat frontal (9)	0.929	121	0.143	0.993	0.360	0.001	3.57	18	32	48
L somatosensory (3)	0.750	186	0.075	0.993	0.360	0.001	3.57	− 42	− 22	38
R frontopolar (10)	0.678	208	0.061	0.994	0.360	0.001	3.55	4	56	30
Digits + words - baseline	L parietal (39)*	0.003	536	0.001	0.011	0.010	0.000	6.29	− 48	− 76	30
R frontopolar (10)*	0.000	951	0.000	0.046	0.010	0.000	5.65	6	60	28
R dor lat frontal (9)*				0.216	0.013	0.000	4.93	18	32	48
L ant cingu (32)*	0.062	251	0.011	0.141	0.011	0.000	5.14	− 16	24	50
L dor lat frontal (9)*				0.972	0.034	0.000	3.70	− 20	34	40
R sup temp (22)*	0.359	109	0.075	0.200	0.013	0.000	5.14	66	− 12	− 16
R prefrontal (11)*	0.003	536	0.001	0.386	0.018	0.000	4.62	2	38	− 12
R parietal (39)*	0.040	291	0.007	0.458	0.020	0.000	4.52	46	− 74	32
L inf temp (20)*	0.771	43	0.247	0.784	0.030	0.000	4.08	− 62	− 14	− 24
L mid temp (48)*	0.757	45	0.237	0.951	0.042	0.000	3.75	− 36	− 18	20
R premotor (6)*	0.919	21	0.421	0.985	0.050	0.001	3.58	22	− 26	70
