# Supplementary material for: You can count on the motor cortex: Finger counting habits modulate motor cortex activation evoked by numbers
Source: Neuroimage. 2012 Feb 15;59-318(4-12):3139–48. doi: 10.1016/j.neuroimage.2011.11.037 (PMC3315027; doi:10.1016/j.neuroimage.2011.11.037)
Supplement: Fig. 1 — Finger counting questionnaire. [file mmc1.pdf]

Supplementary material

Figure 1. Finger counting questionnaire

QUESTIONNAIRE

Thank you for volunteering your time for our research. This questionnaire is very simple and takes only about 2 minutes to complete.

Task 1: Imagine how you would count with your fingers from **1 to 5.** Please type in the numbers (1-5) next to the corresponding fingers of the two hands below.

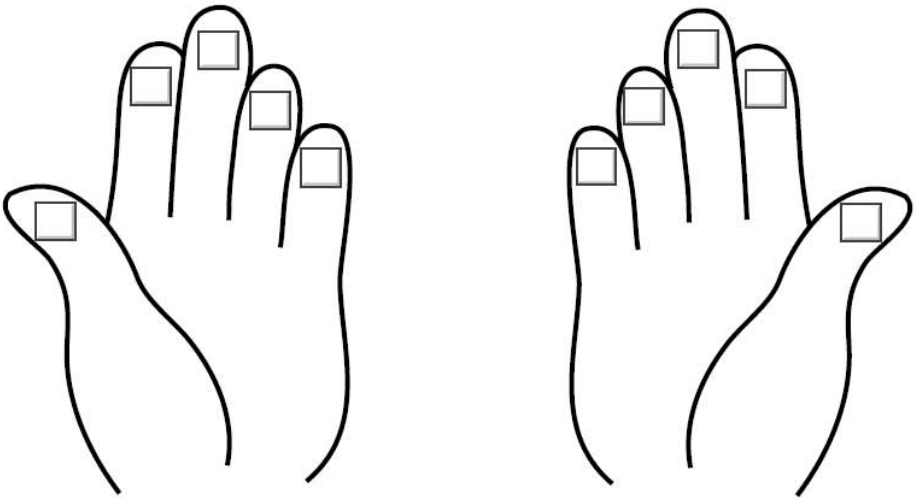

Left **Right**

Task 2: Now please answer these questions:

Imagine how you would count with your fingers from **1 to 10.** Do you still start counting with the same hand? (yes; no; don't know) __________

What is your mother tongue, i.e. which language did you speak first as a child: __________

Did you grow up bilingual, i.e. did you learn two different languages in your childhood at home (Y/N)? __________

Are you right-handed (R) or left-handed (L)? __________

Do you have any immediate relatives, who are left-handed (Y/N)? __________

That's it. Thank you for your participation! Please return the completed questionnaire.
